# Experiences and care needs of children with long COVID: a qualitative study

**DOI:** 10.3399/BJGPO.2023.0143

**Published:** 2024-03-06

**Authors:** Alice Faux-Nightingale, Benjamin Saunders, Claire Burton, Carolyn A Chew-Graham, Glenys Somayajula, Helen Twohig, Victoria Welsh

**Affiliations:** 1 School of Medicine, Keele University, Staffordshire, UK

**Keywords:** child, adolescent, post-acute COVID-19 syndrome, COVID-19, family practice, general practice, primary health care

## Abstract

**Background:**

Long COVID, the patient-preferred term, describes symptoms persisting after an acute episode of COVID-19 infection. Symptoms in children and young people (CYP) can affect daily routine, with broader impacts on education, health-related quality of life, and social activities, which may have long-term effects on health and wellbeing.

**Aim:**

To explore the lived experiences and care needs of CYP with long COVID from the perspectives of CYP with long COVID, their parents, and professionals associated with the care of children and families living with the condition.

**Design & setting:**

CYP and their parent or carer were invited for interview following participation in a cohort study, which recruited the sample from a primary care setting.

**Method:**

Interviews were carried out with four CYP with long COVID (all female, aged 10–17 years); three interviews included a parent. Two focus groups were conducted, which included seven professionals involved with care of CYP or long COVID, from a range of disciplines. Interviews and focus groups were transcribed verbatim, and data analysed thematically using constant comparison techniques.

**Results:**

The three main themes presented are as follows: living with long COVID; uncertainty surrounding long COVID; and seeking help for symptoms.

**Conclusion:**

Long COVID can severely impact the lives of CYP and their families. CYP and their families need to be listened to by professionals and have any uncertainties acknowledged. It is imperative that agencies working with them understand the condition and its impact, and are able to offer support where needed.

## How this fits in

Observational studies describe long COVID in children and young people (CYP), but the impact of the condition on the lives of CYP and their families, including healthcare needs, is not well understood. This research has demonstrated that, while CYP and their families each have their own experiences of long COVID, being heard by healthcare professionals, having symptoms legitimised and uncertainties around long COVID acknowledged during a primary care consultation is essential. While research into prognosis and targeted treatments continues, primary care can support CYP and their families through their journey by listening to their patients, discussing the certainties and uncertainties around long COVID, and understanding the options for further support available in their area.

## Introduction

Nearly all children and young people (CYP) in England have been exposed to the SARS-CoV-2 virus that causes acute COVID-19 infection,^1^ and it is estimated that 25.24% of cases of acute COVID-19 develop into long COVID.^2^ However, studies have frequently observed hospitalised populations where the risk of prolonged symptoms is likely to be higher than in community-treated populations, with one systematic review of CYP with community-treated COVID-19 estimating a 5·1% (95% confidence interval [CI] = 3·6% to 7·0%) risk of prolonged symptoms.^3^

Long COVID is the patient-preferred term used to refer to any symptoms that persist beyond 4 weeks after acute COVID-19.^4^ This encompasses the clinical definitions of ongoing symptomatic (or post-acute) COVID-19, where symptoms continue 4–12 weeks after initial onset, and post-COVID-19 syndrome, where symptoms last more than 12 weeks.^5^ Multiple symptoms have been described in CYP living with long COVID. The commonest include fatigue, breathing difficulties, headaches, myalgia or arthralgia, sleep disturbance, rhinorrhea, coughing, anosmia or dysgeusia, and sensory problems.^6–8^ These symptoms can affect daily routine and school attendance.^9^ This is particularly relevant for CYP as it can impact their education, development and wellbeing, health-related quality of life, and participation in social activities.^6,10–12^

It is important to explore how long COVID is experienced by CYP and better understand how it affects their lives. This understanding can support the development of strategies and treatments to reduce the impact on CYP^13^ and the development of information for use in education, children’s support services, and health and social care.^8^

This study aimed to understand the lived experiences and care needs of CYP with long COVID from the perspectives of CYP with long COVID, their parents, and professionals involved with the care of children and families with the condition.

## Method

A qualitative study nested within the SPLaT-19 project (Symptom Patterns and Life with longer Term COVID-19 in children and young people), which is a mixed-methods study conducted in the UK.^14^ SPLaT-19 aimed to provide a picture of the longer-term effects of acute COVID-19 in CYP aged 8–17 years, residing in the West Midlands. The cohort study identified CYP aged 8–17 years from 40 GP practices within the National Institute for Health and Care Research (NIHR) Clinical Research Network (CRN) West Midlands (WM) and invited them to complete online questionnaires to monitor incidences of COVID-19 and symptoms over a 12-month period. This qualitative study drew participants from the established cohort.

### Patient and public involvement and engagement input

Patient and public involvement and engagement (PPIE) input has been included at every stage of this study from the development to the dissemination. A young person, aged 17 years, is a co-investigator on the study and has contributed to the study design, topic guides, and dissemination of findings. Members of the NIHR CRN WM Young Research Champion’s Group (six CYP, all mid–late teens) were involved with the development of the topic guide for the interviews and focus groups, and later (seven CYP, all mid–late teens) provided insight to the analysis and the dissemination of study findings.

### Participants

#### Interviews

Participants were recruited from the SPLaT-19 cohort study. CYP were invited to participate in an interview if they had reported at least one episode of COVID-19 infection, ongoing symptoms that had lasted longer than 4 weeks within the previous 6 months, and had consented to contact by the research team about the interview study. Sixty-seven emails were sent out to eligible CYP at the email address they provided while participating in the cohort study; this was either a parent’s or a CYP account. The email included information about the study and invited them to participate in an interview, either in-person or virtual. Participants were offered a £20 gift voucher to thank them for their participation. All participants who responded were invited to interview. Participants aged <16 years were asked to be accompanied by a parent during the interview; those aged >16 years were given the option to have a parent present in the interview. Parents who attended the interview were included in discussion and encouraged to talk about their experiences.

#### Focus groups

Professionals with experience of working with CYP, or who worked professionally with CYP or families in long COVID services were identified through the research team’s professional networks, provided with an information sheet about the study, and invited via email to participate in a focus group. Some participants had existing relationships with some members of the study team.

Interview participants aged <16 years were asked to read and sign an assent or consent form with their parents; those aged ≥16 years were asked to complete their own consent form. Focus group participants were asked to complete a consent form. Consent and assent forms were returned to the research team before participation. Consent was verbally confirmed at the start of the interview or focus group.

### Data collection

The interviews aimed to gain an in-depth understanding of participants’ views and experiences of having long COVID, with a particular interest in how long COVID had affected them and their usual activities. Focus groups explored professionals’ experiences of working with CYP and families with long COVID and any information, training, or support they had received to help CYP with long COVID. Topic guides were produced in collaboration with a PPIE group to support the discussion, but the interviewer or facilitator remained flexible within the interview or focus group to respond to topics raised by participants.

Interviews and focus groups were carried out as follows: two interviews were conducted, followed by one focus group, a further two interviews, and, finally, the second focus group. Topic guides were modified iteratively as data generation and analysis progressed, supporting investigation of topics of interest raised in conversations. The first focus group, for example, included questions about experiences of patient use of private health care, and factors that influence the parental experience of supporting CYP with long COVID, based on initial interview findings. Examples of topic guides can be found in the supplementary material.

Interviews ranged between 40 and 70 minutes in length and took place on university premises (three) or virtually, using Microsoft Teams (one) according to the participants’ preferences. Interview length was guided by the participant and included rest breaks as necessary. All interviews were conducted by AFN, an experienced female qualitative researcher.

Focus groups were 76 and 82 minutes long and took place virtually using Microsoft Teams in January and April 2023. Focus groups took place virtually, facilitated by two qualitative researchers, AFN (female) and BS (male, PhD), with GS (a clinician with experience of working with children) also present to document observations.

Interviews and focus groups were recorded with consent. Audio and video recordings of the sessions were sent to a professional transcriber who produced an anonymised transcript for analysis.

### Data analysis

Data collection and analysis were carried out concurrently to support development of themes and subsequent data collection. Data were analysed thematically using the constant comparison method,^15^ each transcript being first analysed separately and then mapped onto other transcripts to compare views and experiences described by participants.

Transcripts were inductively coded manually by AFN, using NVivo software (version 14) to record the codes. Transcripts were coded separately, then codes were mapped on to one another and used to develop preliminary themes, first within sets of interview and focus group data, and then across the whole dataset. Codes and emerging themes were examined and checked by BS, and AFN and BS developed preliminary themes together. Further analysis sessions were held with the whole multidisciplinary team (all authors), to consider preliminary findings in relation to existing theory and clinical practice. Consensus on the final themes was reached through detailed discussion of data in these whole-team meetings where the research team refined the analysis and finalised the key themes.

AFN and BS are health service researchers, while CB, CCAG, HT, GS, and VW are academic GPs who have experience of supporting families and CYP with long COVID; CCAG has conducted previous research reporting lived experiences of people with long COVID. A reflexive approach was taken, in which the researchers acknowledged that their experiences and backgrounds will have influenced the focus and interpretation of the data.

## Results

Four CYP responded to the interview invitation, and all participated in an interview between December 2022 and April 2023. Participant characteristics can be found in Table 1. Three of the four interviews included a parent, and all of those parents participated in the interviews. CYP and parents were encouraged to express their views openly and individually, although joint constructions of their understanding and experiences also took place. Parents generally talked in greater detail about their experiences of pursuing health care and the impact of symptoms on family life, while children discussed personal experiences and the impact of symptoms on school. Participants described a range of symptoms, with three still experiencing persistent symptoms and one fully recovered.

**Table 1.** Interview participant characteristics

Participant identifier	Age at time ofinterview, years	Sex	Ethnic group	Accompanied by parent?
01	14	Female	Mixed: White and Black Caribbean	Yes
02	12	Female	White: English, Welsh, Scottish, Northern Irish, British	Yes
03	10	Female	White: English, Welsh, Scottish, Northern Irish, British	Yes
04	17	Female	White: English, Welsh, Scottish, Northern Irish, British	No

Ten people expressed interest in participating in a focus group, although three people were unable to attend owing to other commitments. To facilitate and enable participation, two focus groups took place, one with five participants, the other with two. Participants worked in a range of settings, were geographically spread across the UK, and each focus group had different professions present. Focus group one was made up of the following professions: two paediatric consultants (both with clinical experience with children with long COVID); one nurse consultant (child and adolescent mental health services); one physiotherapist (adult pain and long COVID services); and a primary school teacher. The second was made up of a physiotherapist (children’s long COVID hub), and a GP. From the focus groups, two consultants and one physiotherapist worked within children’s long COVID services.

The following three key themes were developed from the data: living with long COVID; uncertainty surrounding long COVID; and seeking help for symptoms. Illustrative data extracts are given with participant identifiers.

### Living with long COVID

Participants described multiple symptoms associated with long COVID in CYP. Fatigue and headaches were commonly described in the focus groups and were experienced by all interview participants. Anxiety, breathing difficulties or dysfunctional breathing, and loss or altered smell or taste were also mentioned in both sets of data, although less frequently.

Three CYP used the term ‘long COVID’ to describe their symptoms, and confirmed that they had been given a diagnosis of long COVID or had been referred to long COVID services; the fourth interview participant described experiencing prolonged symptoms since an acute COVID-19 infection, which they had self-managed, but said that they hadn’t considered the symptoms as long COVID until participating in the SPLaT-19 cohort study. Responses and perceptions of long COVID varied according to the severity of their symptoms; for example, those with more severe symptoms used terms such as ‘*horrible*’ (04) to describe their experiences, while those with milder symptoms used descriptors such as ‘*annoying*’ (02).

Three CYP with long COVID reported being unable to attend school for at least some time: two had been fully off-timetable, and the third was going to school when able. These participants explained how this had affected them academically and all described achieving lower grades or worse results than they had achieved before developing prolonged symptoms:

*'I mean my grades are okay* […] *I’ve got a few 5s a few 6s but then in like science* […] *I got a 2 and a 3, do you know what I mean, but I only did three of the six exams.'* (04)

Those who described more severe symptoms described being cut off from friends, leading to loss of friendships and difficulty building new friendships. One participant who had been unable to attend school full-time during a school transition found it hard to make new friends:

*'In form like there’s like no-one that I’m friends with in it so I’m always just like sitting there on my own.'* (01)

CYP described how long COVID symptoms restricted or stopped them from engaging with their hobbies. For one participant, this led to the loss of a place on a competitive team:

*'I like doing bars* […] *it’s really fun to just push myself to different expectations and try different things* […]*, now* [I] *can’t.*' (03)

*'She was selected to go to the* [competitive] *gymnastics squad but that had to stop so we now go to a different class.'* (Parent of 03)

The changes reported were accompanied by a sense of loss and loss of identity, and CYP and parents described how long COVID distanced them from their former lives:

*'We used to go on a lot of walks and I used to do a lot of running at school with my friends, but I don’t do that that much anymore because it’s really tiring.'* (02)

CYP also perceived that there was a lack of knowledge about long COVID within the public. Their narratives included people they knew who were unaware of long COVID or disbelieved the impact it had on them. All interview participants, except the one with mild symptoms, mentioned experiencing judgement or stigma from others about their long COVID, because of lack of awareness of long COVID or a disbelief of its impact:

*'The school and then just general people, like even now, mum is like, some people you can clearly tell that they think you’re putting it on, I was like, I am really not.'* (04)

These concerns caused some CYP to refrain from talking about their long COVID to avoid negative responses, and provoked worry that other people, including healthcare professionals, would not believe their experiences or would mistrust their test results:

*'*[I’m] *scared it will be all my fault for pressing the* [ECG test] *button and no-one will believe me.'* (03)

### Uncertainty surrounding long COVID

Three CYP described seeking medical support for long COVID (the remaining participant had not pursued medical advice). They described feeling that their GPs had been unable to diagnose them with long COVID or offer any suggestion of how they could improve the symptoms, because they did not seem to know enough about it.

*'The GP has mentioned* [long COVID] *and she said it is, probably, but there’s no research on it, we can’t really say one way or the other.'* (Parent of 03)

The uncertainty reported by families was also highlighted in focus groups. Professionals reported that this uncertainty was driven, at least in part, by the presence of other factors that could be contributing to patients’ symptoms. This included the difficulty of disentangling symptoms owing to the effects of the pandemic and associated lockdowns and restrictions, from the symptoms of long COVID, which healthcare professionals suggested may present similarly:

*'It’s very, very difficult to say that the young people that we’re seeing are* […] *afflicted because of the virus infecting them and causing a physiological problem. A lot of it is what they’ve lived through.'* (Paediatric consultant, long COVID clinic)

Professionals considered differential diagnoses to explain the symptoms experienced by the children they worked with; for example, neurodiversity was suggested to be a contributing factor to symptoms such as fatigue, while deconditioning secondary to pandemic restrictions was suggested to have potentially contributed to respiratory difficulties:

*'The biggest group are the ones who actually their fatigue is because of their anxiety and their low mood or their neurodiversity.'* (Paediatric consultant, long COVID clinic)

*'Being neurodiverse or being anxious or being depressed are all very exhausting things and so* […] *they’re now turning up in the post-COVID clinics.'* (Paediatric consultant, long COVID clinic)

Two parents described times when healthcare professionals had made comments to them about their children’s symptoms being related to these other factors. However, alternative explanations for symptoms, particularly suggestions that the symptoms were consequences of the pandemic, were not fully accepted by parents, who suggested that they did not entirely explain their child’s experiences:

*'At the hospital they said that it’s down to COVID as a whole, being in lockdown* […] *perhaps in the short term that could have been an explanation but not two years in. And we’ve tried everything to try and build it back up as they’ve suggested and it’s just not improving, so I am a little sceptical on that one.'* (Parent of 03)

Parents who participated in the interviews described the emotional toll of this uncertainty and talked about feelings of guilt, which they felt because they were unsure whether their actions had caused or exacerbated their child’s illness or if they were helping them get better:

*'Am I doing the right thing? I don’t know. I really, really don’t know.*' (Parent of 03)

They said that they would like more information and guidance to help them help their child:

*'I don’t know if there is any treatment that could potentially help her presentation of symptoms, if there was I would most definitely want to seek it out.'* (Parent of 02)

### Seeking help for symptoms

Three CYP described trying to access medical support for long COVID, and one had attended a long COVID clinic; but all these participants talked about difficulty accessing long COVID services, with location and availability of appointments the biggest barriers to accessing care. One parent, who attended a long COVID clinic, described difficulties navigating public transport with their child when they were struggling with fatigue:

*'We went to the long COVID clinic in* [place] *in August* […] *It took quite a while to wake* [01] *and we ended up by getting a taxi all the way there* [because] *I don’t drive.*' (Parent of 01)

Another parent described having to turn down an appointment at a long COVID clinic because it was too far away for her to access. She said that she had been offered that appointment at that clinic because it was the only appointment available to her, despite there being nearer long COVID clinics:

*'I was offered to go to one* [long COVID clinic] *in* [place ~3hr drive away] *because they said that that was the only one that they had. So we politely, we declined that and they’ve been seeing her at the hospital*.' (Parent of 03)

Patients’ difficulty accessing long COVID and other children’s services, was also mentioned in focus groups, although these difficulties were associated with a lack of understanding about the services and referral routes in place to help children with long COVID.

*'A lot of our families have been passed around the houses, they haven’t had an easy journey to get to us often because people didn’t know we existed* […] *we need to make the pathways smoother so, that the likes of* [GP participant] *knows how these pathways work.'* (Paediatric physiotherapist, long COVID clinic)

Professionals, however, described other factors that might affect access to long COVID services, such as funding and recruiting healthcare professionals to some long COVID clinics.

*'As far as the government is concerned, COVID is over* […] *there is going to be no funding after next year, but post-COVID isn’t going anywhere.'* (Paediatric physiotherapist, long COVID clinic)

When unable to find help, interview participants described going to other places for information or treatment or trying to help themselves in a trial-and-error fashion. They described trying private health care, acupuncture, and reiki, or visiting online long COVID forums for emotional support and advice, trying treatments suggested by other parents, and the benefits they felt from accessing support in these ways.

*'I went to this woman who’s like a spiritual person and she did like reiki* […] *and after I was like, I feel great now.'* (04)

*'I look online, I get things from — get quite a lot from the ME Association, they’ve got quite a lot information on their website.* […] *Long COVID* [Kids] *they basically concentrate on basically children and teenagers, teenagers and that, they do like chat groups* […] *I get quite a lot of information, things like that, what people have tried, what’s worked.'* (Parent of 01)

## Discussion

### Summary

This study has demonstrated the variety and complexity of lived experiences and care needs of CYP with long COVID. Families described the impact of long COVID on the education, recreation, and sense of identity of the affected young person, and described experiencing uncertainty and stigma. While some professionals suggested that it can be difficult to disentangle symptoms of long COVID from other factors, including restrictions at the height of the pandemic, professionals highlighted the support that long COVID services can offer CYP with long COVID, although access to these services is limited.

### Strengths and limitations

Interviewing CYP with direct experience of long COVID produced rich data that supported a holistic view of the lived experience of CYP with long COVID and challenges that some face when accessing health care. The multidisciplinary team involved in analysis, including academic GPs and social scientists, was a strength and contributed to a broader understanding of the findings.

Interviewing four participants, three parents, and seven participants in the focus groups resulted in a dataset from a heterogeneous population, including a broad age range for interviewees and a mix of professional backgrounds. While each participant described their unique experiences, overall themes and descriptions were similar across the participant groups. It is possible that additional interviews with people of different backgrounds will find different themes, for example, including greater diversity in ethnic group or professional groups. Although data saturation may not have been achieved, our dataset has produced rich findings from a range of participants of different ages and professional backgrounds that are of immediate utility to frontline clinicians and of value to the development of long COVID health and care support.

While the study included every participant who responded to the interview invitation and nearly every focus group participant who expressed interest, the low response rate from participants resulted in a low number of participants. The study identified little public knowledge about long COVID and also found stigma associated with the condition; both may have affected recruitment. For the focus groups, industrial action and pressures across teaching and the NHS at the time of data collection are likely to have affected recruitment and oriented conversation towards funding and strained services.

### Comparison with existing literature

Our research has highlighted the impact that long COVID can have on CYP, in terms of physical symptoms and broader lived experience. While experiences of CYP show similarity to those of adults with long COVID,^16^ our study has drawn attention to the unique social and educational circumstances that affect children. Time away from school was common among participants, corroborating existing studies.^9,17^ However, previous research has not explored CYPs' views on their experiences, and our results showed that the sudden, widespread loss of social and recreational activities, which occurred as a result of long COVID, impacted on CYPs’ sense of identity and social connections.

Inclusion of parents provided further insight into the way that long COVID affects families, and the places they seek information and support. High levels of stress have been observed in parents of children with long COVID;^18^ in the present study, uncertainty and lack of knowledge about long COVID were associated with parental guilt, as they were unsure of whether their actions were positively or negatively affecting their child’s health. Parents described seeking support from healthcare settings and visited other sources, such as internet forums, for emotional support, advice, and treatment suggestions.^19–21^ Parents raised logistical difficulties with accessing appointments, both in terms of availability of appointments in long COVID clinics, similar to existing research,^22^ and the practicalities of attending appointments such as distance or difficulties accessing locations on public transport. These findings resonate with the Dixon-Woods candidacy model of access to services, particularly their discussion about the impact of the pandemic in reducing the permeability of health services.^23^ The model highlights how access to healthcare services is not only associated with the supply of services, but also the ability of people to access them, whether owing to effort or resources, such as technology, as in the model, or barriers such as public transport, discussed in the present study. Where permeability is low and barriers to access are in place, families can be cut off from accessing the care they need, as described by participants in this study, and this can have implications for health.

### Implications for practice

Although knowledge is still emerging about how long COVID affects CYP, this study has highlighted the significance of the clinician–patient relationship and has shown how support from healthcare professionals is an essential component of patients’ healthcare journeys. A key role of the GP is to support families through their experiences, and it is important that they listen to CYP and their families, understand their experiences and perspectives,^24,25^ and make sure they feel listened to and believed.^16,25,26^ Person-centred care is the cornerstone of this approach,^27^ exploring ideas, concerns, and expectations in every consultation that includes the CYP. Confirming what is known about long COVID and acknowledging uncertainties with parents can help with much of the uncertainty experienced by families, as could signposting to reputable sources of information, considering literature and information that patients may have brought with them to the consultation, or working with families to explore other sources of support, and ensuring that any remedies sought are safe. While this could appear difficult, information that patients want may be relatively basic. When the results of this study were discussed with a PPIE group they suggested the following simple topics: long COVID can exist in children; long COVID is not infectious; everyone is affected differently; and everyone recovers at different rates. This information may ensure that families do not leave feeling dismissed and could also counter some misunderstandings and stigma about long COVID among family members,^28^ a common challenge reported for adults living with long COVID.^29^ Another request was for more information about what would happen if healthcare support was sought for their symptoms and for information about what healthcare professionals could do to help; given local variation, it is advisable for clinicians to understand the referral processes in their local area to be able to advise patients accordingly.^25,30^ It should be noted, however, that the families in this study were approaching their GP about symptoms during the early to middle phases of the pandemic, and the situation may be different for CYP who seek medical support now.

In conclusion, this qualitative study has highlighted the experiences of long COVID from the perspectives of CYP, parents, and professionals. CYP and their families need to be listened to by professionals and be able to explain their symptoms and the impact on everyday life. Long COVID can severely impact the lives of CYP and their families and it is imperative that agencies working with them have an understanding of the condition and its impact, and can offer support in all areas of need.

Uncertainties around long COVID in CYP and their families are mirrored by uncertainties in professionals, which can lead to CYP feeling that they are not believed. GPs have an important role in listening to CYP with long COVID, supporting them, and referring to long COVID services if available.^31^

## Data

The dataset relied on in this article is not available as participants did not consent to sharing their data in this way.
